# Gastrointestinal clear cell sarcoma, AKA malignant gastrointestinal Neuroectodermal tumor: an uncommon entity in a young patient presenting with Anemia, Intraabdominal mass and subsequent liver metastasis

**DOI:** 10.1093/omcr/omaf028

**Published:** 2025-05-28

**Authors:** Sara Salehiazar, Sava Grujic

**Affiliations:** Pathology and Laboratory Medicine, Harbor-UCLA Medical Center, 1000 W Carson St, Torrance, California 90502, United States; Pathology and Laboratory Medicine, Harbor-UCLA Medical Center, 1000 W Carson St, Torrance, California 90502, United States

**Keywords:** gene fusion EWSR1-ATF1, next-generation sequencing (NGS), EWSR1 (22q12.2), malignant gastrointestinal neuroectodermal tumor, gastrointestinal clear cell sarcoma

## Abstract

Gastrointestinal clear cell sarcoma is a rare tumor with neuroectodermal differentiation that affects the gastrointestinal tract and involves gene fusion translocations of EWSR1. These tumors predominantly occur in young adults and often display aggressive behavior, with metastases to lymph nodes and the liver. Histologically, the tumor comprises uniform round cells with round nuclei and pale eosinophilic or clear cytoplasm. It exhibits variable mitotic activity and demonstrates positive immunohistochemical staining for S100 and SOX10, while specific melanocytic markers are negative. Currently, no tailored chemotherapy regimen has been identified for this entity. Due to the limited number of reported cases, effective management strategies remain unclear. Here, we present the case of a young adult patient diagnosed with CCS/GNET using immunohistochemistry. The diagnosis was confirmed by next-generation sequencing (NGS), which detected the characteristic EWSR1-ATF1 gene fusion, and liver metastases were identified during follow-up.

## Introduction

Gastrointestinal clear cell sarcoma is a rare mesenchymal tumor with neuroectodermal differentiation that affects the gastrointestinal tract and involves gene fusion translocations of EWSR1. Histologically, the tumor typically consists of uniform round cells with round nuclei and pale eosinophilic or clear cytoplasm, although areas with spindle cell morphology may also be present. Tumor cells are often separated by fibrous septa, and multinucleated osteoclast-like giant cells are observed in approximately half of the cases. Mitotic activity is variable. Positive immunohistochemical staining for S100 and SOX10 is characteristic, while melanocytic markers such as Melan A and HMB45 are, by definition, negative. Staining for synaptophysin and CD56 is commonly observed but may be patchy. In this report, we present a case of jejunal CCS and discuss its clinicopathological, immunohistochemical, and molecular features, as well as the differential diagnoses of CCSs.

## Case presentation

**Figure 1:**
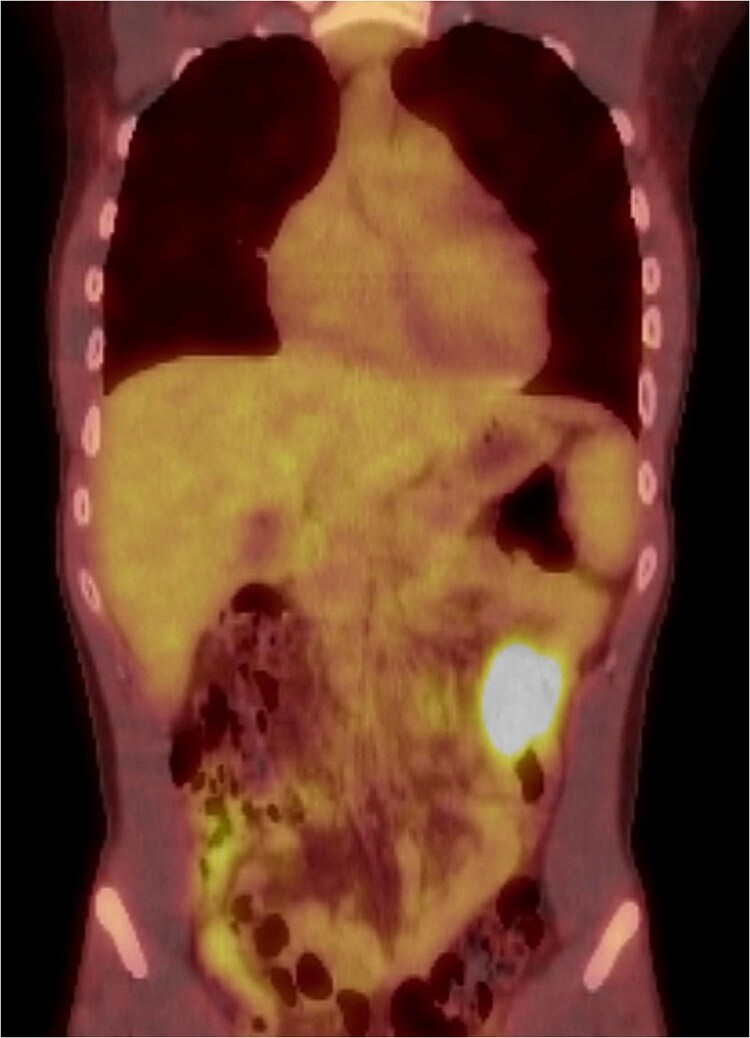
PET/CT scan: Showing focal wall thickening of small bowel loop in the left abdomen with increased FDG uptake (SUV max 8.7).

Our patient was a young adult who was previously healthy, a non-smoker, and a social drinker, with no history of drug use. His family history was non-contributory. The patient presented with progressive fatigue, weight loss, and significantly low hemoglobin levels (4.6 g/dl). Initial investigations, including endoscopy and colonoscopy, were conducted to evaluate a suspected malignancy as the source of bleeding, but no abnormalities were identified. The patient underwent multiple upper gastrointestinal endoscopies, all of which were unremarkable and failed to reveal any abnormalities. Due to persistent concerns about malignancy, a PET/CT scan was performed. The scan revealed focal wall thickening of a small bowel loop in the left abdomen with increased FDG uptake (Fig. 1).

**Figure 2:**
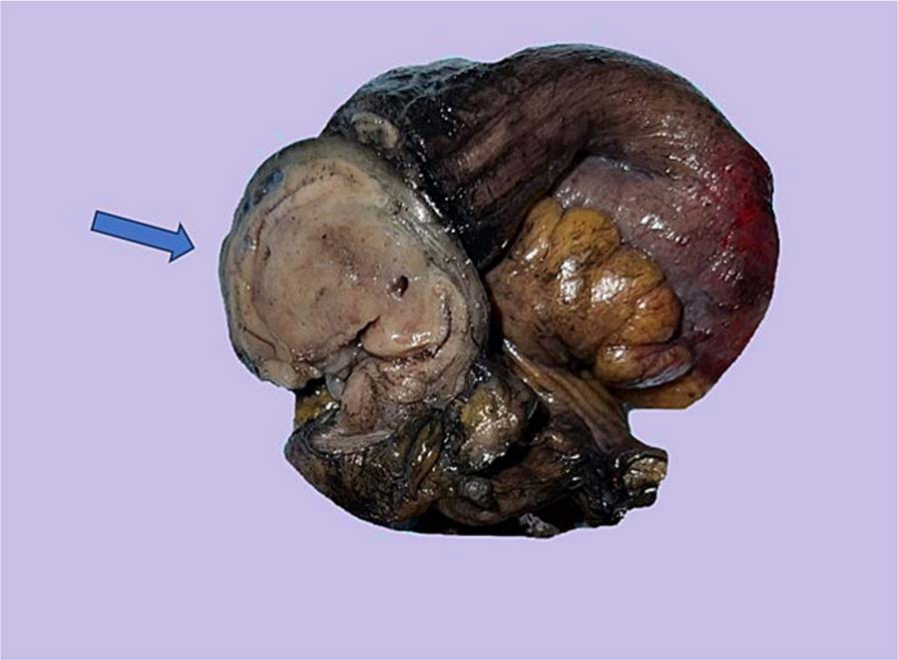
Gross appearance: Show well circumscribed solid tan-pink cut surface.

Subsequently, the patient underwent diagnostic laparoscopy with segmental small intestinal resection. Gross examination revealed a 5 cm annular mass involving the full thickness of the jejunal wall (Fig. 2). On cross-section, the mass appeared solid and tan-white, with an unremarkable mucosal surface.

**Figure 3:**
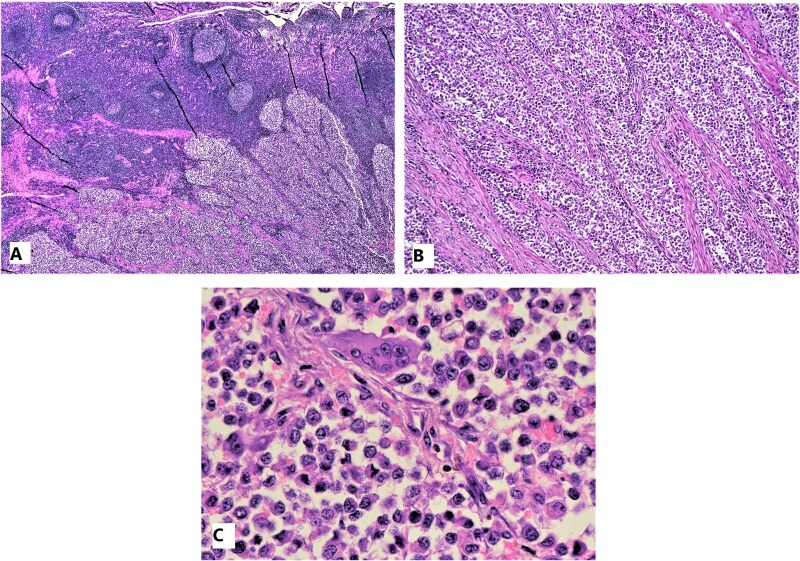
Microscopic pictures: Clusters of malignant cells show upward invasion of the mucosa (A), clusters of malignant cells separated by fibrovascular cores (B), high-power view of tumor cells and a single multinucleated giant cell (C).

A low-power histologic exam shows clusters and sheets of round epithelioid cells separated by fibrovascular bands. The tumor is mainly submucosal and only focally invades mucosa (Fig. 3A). The intermediate power exam shows sheets and clusters of malignant cells separated by fibrovascular cores. Clear cytoplasmic appearance can be appreciated at this magnification (Fig. 3B). High power exam shows the cells with large round nuclei, occasional prominent nucleoli and light eosinophilic to clear cytoplasm. Mitotic figures were inconspicuous. Osteoclast-like multinucleated giant cell can also be seen (Fig. 3C).

**Figure 4:**
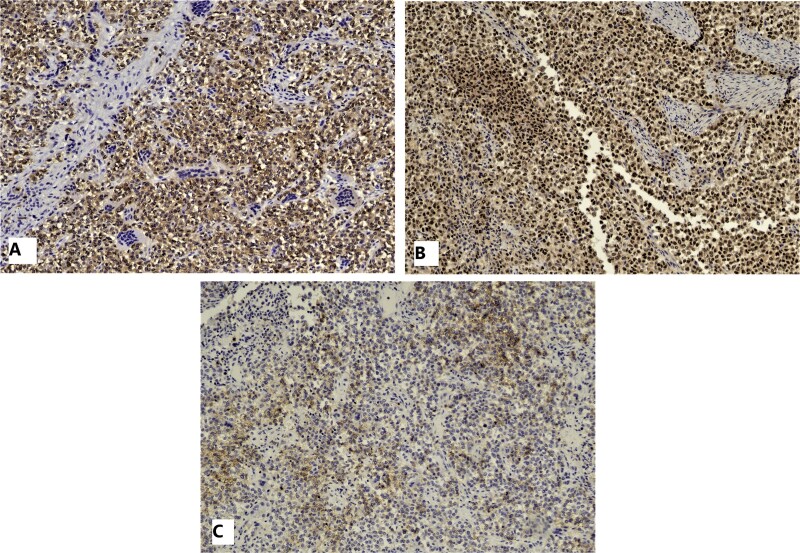
IHC: Tumor cells are strongly and diffusely positive for S-100 (A), Sox-10 (B), and patchy positive for CD56 (C).

Immunohistochemistry demonstrated nuclear and cytoplasmic positivity for S100 (Fig. 4A), positive nuclear staining with SOX10 (Fig. 4B), and patchy membranous staining with CD56 (Fig. 4C). The tumor was negative for melanocyte-specific markers (Melan A and HMB-45), SALL4, Synaptophysin, and CD117.

**Figure 5:**
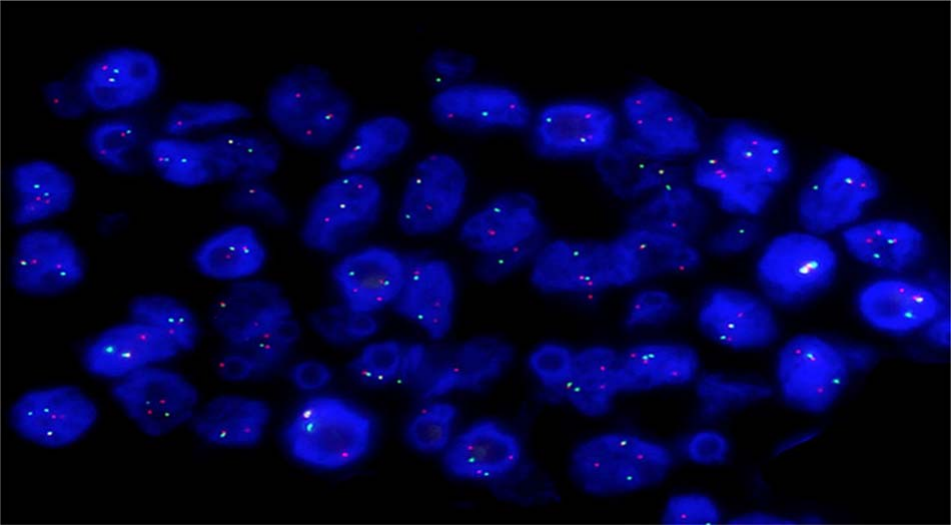
Molecular study with FISH analysis: Positive FISH result for EWSR1 (22q12.2) rearrangement.

Molecular studies included FISH analysis, which confirmed an EWSR1 (22q12.2) rearrangement (Fig. 5). Next-generation sequencing (NGS) identified an EWSR1-ATF1 chromosomal rearrangement and tumor mutational burden of 4.2 mutations/MB (low).

**Figure 6:**
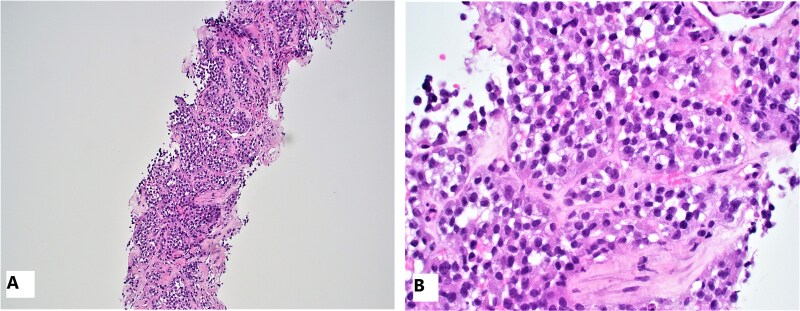
Microscopic image of the liver biopsy mass: H&E shows similar microscopic features of clear cell sarcoma, which shows clusters of uniform tumor cells with clear cytoplasm that replace the liver parenchyma, 20x (A) and 40x (B).

Post-operatively, the patient did well with improved symptoms and a hemoglobin level that increased to 14.7 g/dl. The patient was presented at a multidisciplinary tumor board conference, which concluded that no adjuvant therapy is indicated at this time due to the negative lymph node status, and frequent surveillance was recommended. Approximately five months later, during routine surveillance imaging, a whole-body scan was negative except for a newly identified peripherally enhancing lesion in the posterior right hepatic lobe, measuring 1.5 cm, which was not on prior examinations exams. MRI with contrast was performed to better visualize the liver lesion, revealing three small, indeterminate, ill-defined lesions in the posterior right hepatic lobe, measuring up to 13 mm, with surrounding perfusion changes concerning for metastatic disease. The patient underwent a CT-guided liver biopsy. The initial biopsy was negative for metastatic tumor. The second biopsy showed the tumor with histomorphologic features identical to those observed in the jejunal resection (Fig. 6A and B).

**Figure 7:**
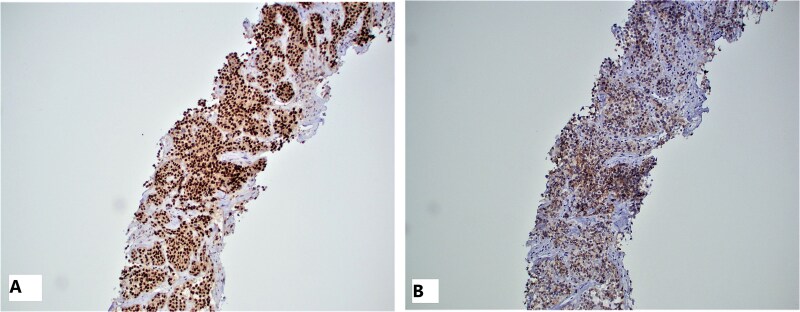
IHC of the liver mass: Tumor cells are strongly positive for Sox-10 (A) and patchy positive for CD56 (B).

The immunohistochemical profile performed and showed positive Sox-10 (Fig. 7A), S-100, focally positive CD56 (Fig. 7B) and negative synaptophysin. The histology and IHC confirmed the diagnosis of metastatic malignant gastrointestinal neuroectodermal tumor.

## Discussion

Gastrointestinal clear cell sarcoma (CCS) of the gastrointestinal tract is a rare entity first described in 2003 [1]. To the best of our knowledge, fewer than 100 cases have been reported in the English literature [1]. This tumor is most commonly found in young adults, with more than 60% of patients being under the age of 45 and a median age of diagnosis of 35 years [2]. Patients typically present with symptoms such as abdominal pain, vomiting, abdominal distension, weight loss, and anemia [2]. The exact etiology of CCS remains unknown; however, some case reports suggest that a history of radiotherapy may contribute to its pathogenesis [3]. Exposure to chemotherapy or radiotherapy during childhood for conditions like neuroblastoma, hepatoblastoma, acute lymphoblastic leukemia, or Ewing’s sarcoma has been suggested as a potential trigger for these tumors [4].

CCS most commonly arises from the wall of the small intestine and less frequently from the walls of the stomach, colon, or peritoneum [5]. At the time of diagnosis, most cases present with regional lymph node involvement or distant metastases, or patients develop recurrence following surgical excision. The liver, peritoneum, and omentum are the most common metastatic sites [1]. Consequently, the clinical course of gastrointestinal CCS appears more aggressive than that of clear cell sarcoma of the soft tissue [5]. As a malignant tumor originating in the interstitium of the gastrointestinal tract wall, it must be differentiated from gastrointestinal stromal tumors (GISTs), synovial sarcoma, and malignant peripheral nerve sheath tumors [6]. Histologically, CCS can be misinterpreted as other non-epithelial gastrointestinal tumors or metastatic tumors, such as seminoma. Awareness of the diagnostic criteria is crucial to avoid misdiagnosis. Strong S-100 staining in most tumor cells is atypical for GISTs and helps exclude leiomyosarcoma. Furthermore, GISTs typically express CD117 (c-Kit), DOG1, and CD34, which are absent in CCS, and c-Kit fusion is a common feature in GISTs [7]. Another important differential diagnosis is clear cell sarcoma of soft tissue; however, CCS lacks melanocytic markers such as HMB-45 and Melan-A, which are commonly found in the soft tissue variant [7]. Identifying the t(12;22)(q13;q12) translocation through FISH analysis further aids in distinguishing CCS from other tumors. Microscopically, CCS is characterized by uniform epithelioid cells arranged in various patterns, including solid formations, nests, and pseudopapillary architectures. Osteoclast-type giant cells are frequently observed, but melanin pigment or evidence of melanocytic differentiation is usually absent [4]. The tumor cells are positive for S-100 and SOX-10, with at least focal positivity for CD56. However, melanocytic markers such as Melan-A and HMB-45 are consistently negative [3]. Diagnosis is challenging due to the absence of specific clinical manifestations and the limitations of gastrointestinal endoscopy, making imaging a critical tool for detecting abnormalities. CCS should be considered when significant enhancement of gastrointestinal tumors is observed in the venous phase, particularly if accompanied by liver or lymph node metastases [8]. MRI, with its high-resolution and functional imaging capabilities, offers unique advantages, often providing early indications of disease and prompting timely biopsy. However, definitive diagnosis of CCS relies primarily on pathological evaluation [8].

## Conclusions

CCSs are highly aggressive malignancies with a high likelihood of local recurrence and distant metastasis, posing significant challenges to patient management and outcomes. Nearly 50% of patients succumb to the disease within three years of diagnosis, reflecting its aggressive clinical course and the limitations of current treatment approaches. The cornerstone of diagnosis lies in the identification of genetic alterations, particularly the hallmark EWSR1-ATF1 or EWSR1-CREB1 gene fusions. These fusions are pivotal for distinguishing CCS from other morphologically similar malignancies. Advanced molecular diagnostic techniques such as fluorescence in situ hybridization (FISH), reverse transcription polymerase chain reaction (RT-PCR), and next-generation sequencing (NGS) have revolutionized diagnostic accuracy, enabling pathologists to confirm CCS with high specificity. Despite these advancements, the rarity of the tumor and its histopathological overlap with other neoplasms often lead to delayed or misdiagnosed cases, which further complicates patient management and impacts prognosis. Early and accurate diagnosis plays a critical role in guiding treatment decisions and improving patient outcomes. Given the aggressive nature of CCS, identifying the genetic signature not only confirms the diagnosis but also facilitates the development of tailored surveillance and therapeutic strategies. Timely detection and intervention can significantly impact the prognosis, providing a potential window for more effective disease control and better quality of life for affected patients.

Unfortunately, there is no consensus or standardization of adjuvant therapy for CCS. The effectiveness of chemotherapy and radiotherapy remains limited, with most cases showing minimal or no benefit. Although tyrosine kinase inhibitors have been administered postoperatively in some cases, their efficacy has not been extensively studied. The lack of robust clinical evidence highlights the need for collaborative research and clinical trials to explore novel therapeutic strategies. Targeted therapies, immunotherapy, or other precision medicine approaches may hold promise for addressing the limitations of current treatments.

As CCS continues to be a diagnostic and therapeutic challenge, there is an urgent need for increased awareness among clinicians and pathologists. Multidisciplinary collaboration, incorporating molecular diagnostics, imaging, and advanced surgical techniques, can aid in better understanding and managing this rare malignancy. Future research efforts should focus on elucidating the underlying biology of CCS, identifying actionable targets for therapy, and standardizing treatment protocols to improve survival rates and overall patient care.

## Data Availability

Not applicable.

## References

[1] Nachiappan M, Srikantaiah GD, Gadiyaram S. Clinical, pathological, and genetic profile of clear cell sarcoma-like tumour of jejunum: report of a rare aggressive tumour of small bowel. Clin J Gastroenterol 2021;15:107–11. 10.1007/s12328-021-01554-9 34792784

[2] Park SY, Seo JW. Clear cell sarcoma-like tumour of jejunum: report of a rare aggressive tumour of small bowel. J Korean Soc Radiol 2023;84:1169–75. 10.3348/jksr.2022.0163 PMC10585088 37869114

[3] Wang J, Thway K. Clear cell sarcoma-like tumor of the gastrointestinal tract; an evolving entity. Arch Pathol Lab Med 2015;139:407–12. 10.5858/arpa.2013-0547-RS 25724038

[4] Sonai MK, Rastogi S, Madhusudhan KS. et al. Clear cell sarcoma like tumor of gastrointestinal tract: experience of three cases and review of literature. Indian J Pathol Microbiol 2024;63:90–5. 10.4103/IJPM.IJPM_195_19 32031130

[5] Askan G, Kombak FE, Seven IE. et al. Clear cell sarcoma-like tumor of the gastrointestinal tract. J Gastrointest Canc 2018;50:651–6. 10.1007/s12029-018-0069-4 PMC7469948 29623567

[6] Okada T, Hirano Y, Ishikawa S. et al. A long-term survivor of clear cell sarcoma-like tumor of the gastrointestinal tract with liver metastasis: a case report. Surg Case Rep 2020;6:1–9. 10.1186/s40792-020-01028-z 33025168 PMC7538498

[7] Friedrichs N, Testi MA, Moiraghi L. et al. Clear cell sarcoma-like tumor with osteoclast-like Giant cells in the small bowel: further evidence for a new tumor entity: 2005. Int J Surg Pathol 2005;13:313–8. 10.1177/106689690501300402 16273186

[8] Liao S, Wang X, Li J. et al. Clinical presentation and imaging characteristics of clear cell sarcoma-like tumour of the gastrointestinal tract with liver metastasis: a case description. Quant Imaging Med Surg 2021;11:4690–4. 10.21037/qims-21-186 34737937 PMC8511728

